# TiN/Ti/HfO_2_/TiN memristive devices for neuromorphic computing: from synaptic plasticity to stochastic resonance

**DOI:** 10.3389/fnins.2023.1271956

**Published:** 2023-09-19

**Authors:** David Maldonado, Antonio Cantudo, Eduardo Perez, Rocio Romero-Zaliz, Emilio Perez-Bosch Quesada, Mamathamba Kalishettyhalli Mahadevaiah, Francisco Jimenez-Molinos, Christian Wenger, Juan Bautista Roldan

**Affiliations:** ^1^Departamento de Electronica y Tecnologia de Computadores, Facultad de Ciencias, Universidad de Granada, Granada, Spain; ^2^Materials Research Department, IHP-Leibniz-Institut fuer innovative Mikroelektronik, Frankfurt an der Oder, Germany; ^3^Mathematics, Computer Science, Physics, Electrical Engineering and Information Technology Department, Brandenburg University of Technology Cottbus-Senftenberg (BTU), Cottbus, Germany; ^4^Center for Research in Information and Communication Technologies (CITIC), Andalusian Research Institute on Data Science and Computational intelligence (DaSCI), University of Granada, Granada, Spain

**Keywords:** resistive switching devices, neuromorphic computing, synaptic behavior, spike timing dependent plasticity, stochastic resonance

## Abstract

We characterize TiN/Ti/HfO_2_/TiN memristive devices for neuromorphic computing. We analyze different features that allow the devices to mimic biological synapses and present the models to reproduce analytically some of the data measured. In particular, we have measured the spike timing dependent plasticity behavior in our devices and later on we have modeled it. The spike timing dependent plasticity model was implemented as the learning rule of a spiking neural network that was trained to recognize the MNIST dataset. Variability is implemented and its influence on the network recognition accuracy is considered accounting for the number of neurons in the network and the number of training epochs. Finally, stochastic resonance is studied as another synaptic feature. It is shown that this effect is important and greatly depends on the noise statistical characteristics.

## 1. Introduction

Memristive devices are considered promising alternatives both for stand-alone and embedded non-volatile memory circuits (Yu, 2022). Other applications are connected to data security (Carboni and Ielmini, 2019; Wen et al., 2021; Yang et al., 2021) and mobile communications (Lanza et al., 2022). However, the most interesting use of these emerging devices is linked to the hardware implementation of artificial neural networks in the context of neuromorphic engineering (Allen et al., 1989; Zhu et al., 2023). In this latter case, the memristive device outstanding features to mimic the behavior of biological synapses (conductance potentiation and depression, spike-timing dependent plasticity (STDP), spike-rate dependent plasticity (SRDP), paired-pulse facilitation (PPF), vector matrix multiplication (VMM) in crossbar arrays, etc.) play an essential role (Alibart et al., 2013; Merolla et al., 2014; Prezioso et al., 2015; Ambrogio et al., 2018; Zidan et al., 2018; Sebastian et al., 2020; Hui et al., 2021; Pérez-Bosch Quesada et al., 2021; Yu et al., 2021; Roldan et al., 2022; Zhu et al., 2023).

Among the variety of memristive devices, those based on filamentary conduction are very common. In this case, the device operation is facilitated by the formation and destruction of nanometric filaments that short the metal electrodes grown at both sides of a dielectric layer (Guy et al., 2015; Huang et al., 2017; Dirkmann et al., 2018; Pérez et al., 2019; Aldana et al., 2020b; Funck and Menzel, 2021). The devices we study in this manuscript show this type of filamentary operation; they are known as resistive random access memories (RRAMs) or resistive memories. RRAMs show exceptional general characteristics such as fast speed (< 10 ns), large (high resistance state, HRS/low resistance state, LRS) ratios (>100), very low switching energy (<0.1 pJ), and high scalability (they are CMOS technology compatible). From the commercial viewpoint, Fujitsu has low-power 8-Mb stand-alone RRAM chips (they operate at 1.6 V with an average read current of 0.15 mA), suitable for IoT applications (Lanza et al., 2022; Fujitsu, 2023); Sandisk/Toshiba reported stand-alone RRAM memory chips with 32 GB (24 nm node technology) (Liu et al., 2013; Lanza et al., 2022).

Neuromorphic engineering using resistive memories enables new computing schemes where the output is generated and stored on-site without having to move data in and out. In this respect, the limitations linked to the Von Neumann's bottleneck are avoided (Sebastian et al., 2020; Lanza et al., 2022). In addition to the improvement in connection to Von Neumann's bottleneck, an advance can also be achieved in terms of overcoming the hurdles linked to the memory wall (i.e., the steadily growing performance gap between the different types of memory and the microprocessors) (Tang et al., 2019). The role of resistive memories in this new computing paradigm (Yu et al., 2011, 2021; Zheng and Mazumder, 2019; Sebastian et al., 2020; Zhao et al., 2020; Romero-Zaliz et al., 2021; Roldan et al., 2022) is vital to save time and reduce power consumption in artificial intelligence solutions since CMOS-based solutions are not power- and area-efficient. In this respect, as it is shown below, a single device can successfully mimic many features of biological synapses (Sebastian et al., 2020; Yu et al., 2021; Chen et al., 2022; Ismail et al., 2022). Hence, the role of resistive memories in conventional neural networks consists in implementing the synaptic weights. These weights are obtained by means of a quantization process, employing a multilevel conductance approach for the memristive device operation (Milo et al., 2016; Perez et al., 2017; González-Cordero et al., 2019; Sokolov et al., 2019; Ren et al., 2020; Ha et al., 2022; Roldán et al., 2023a).

There are two main types of neural networks behind AI applications: artificial neural networks (ANNs) and spiking neural networks (SNNs). For ANN, information is encoded with continuous values. They can reach high data recognition accuracy with two or more layers of non-linear neurons connected by synaptic weights (Sebastian et al., 2020). Thus, large networks with thousands of synapses can be implemented (Yu et al., 2021). On the contrary, information is coded with time-dependent spikes in SNNs, this feature reduces power consumption in comparison to ANNs (Zheng and Mazumder, 2019). Several features distinguish ANNs and SNNs; among them, the most remarkable are the following: (a) the manner in which information is encoded (in ANNs real-value activations are employed to convey information, while in SNNs a series of time-dependent spikes are used), (b) ANN related neurons do not have memory; however, they do have in SNNs, and (c) ANN output (e.g., feed-forward ones) is not time dependent, while it is in SNNs (Zheng and Mazumder, 2019). In SNNs, it is feasible the use of algorithms able to adapt and evolve with time; they have an asynchronous nature that leads to a high system scalability and general efficiency since no synchronization mechanisms are needed (Ezra Tsur, 2022). In this context, we have analyzed SNNs implementing the device STDP behavior as the learning rule (a temporally asymmetric form of Hebbian learning induced by tight temporal correlations between the spikes of pre- and postsynaptic neurons). In particular, the role of variability in the STDP features has been comprehensively studied by considering different SNNs and characterizing their recognition accuracy for an input of standard image dataset. We considered different number of neurons and different training conditions (e.g. varying the number of epochs).

One of the representative biological synaptic features that can be mimicked by memristive devices, in addition to those described above, is stochastic resonance (SR), that is known to be essential in sensory neurobiology (Douglass et al., 1993; Vázquez-Rodríguez et al., 2017). The term SR was first used in 1980 in an explanation of the periodic occurrence of ice ages on Earth (Benzi et al., 1981). Experimentally, SR was seen in 1983 after a laboratory demonstration in Schmitt triggers (Fauve and Heslot, 1983). SR is applied to describe any phenomenon where the presence of input noise (both internal or external) in a non-linear system ends up with a better system response to certain input signal in comparison with the lack of noise (Samardak et al., 2009; Stotland and Di Ventra, 2012). It does not take place in linear systems (McDonnell, 2008). The word resonance comes from a comparison to systems that show a maximum signal-to-noise ratio or output response for some resonance frequencies. In this case, SR would be represented by a maximum output response for a certain noise intensity.

We have studied here stochastic resonance in HfO_2_-based memristors in addition to other synaptic characteristics. To do so, several types of noise sources were employed (Gaussian, uniform, etc.) whose standard deviations were swept in the study (from 50 mV to 150 mV). Our experiments correspond to the first case studies in SR where the systems (the devices) were driven by a combination of a periodic single frequency input signal (ramped voltages to drive conventional resistive switching (RS) operation) and a broadband noise (McDonnell, 2008). In our study, the existence of set and reset processes poses the presence of thresholds in the device operation that allows to observe SR effects. In this respect, we are facing a non-linear device with thresholding (linked to set and reset events) where SR (calculated as the resistance ratio between the OFF and ON states) can be observed and used for the improvement of the output signals in several applications (Mikhaylov et al., 2021). We took into consideration progressive switching events and the inherent RS variability (Pérez et al., 2019; Perez et al., 2023; Roldán et al., 2023b).

## 2. Device fabrication and measurement setup

**Figure 1:**
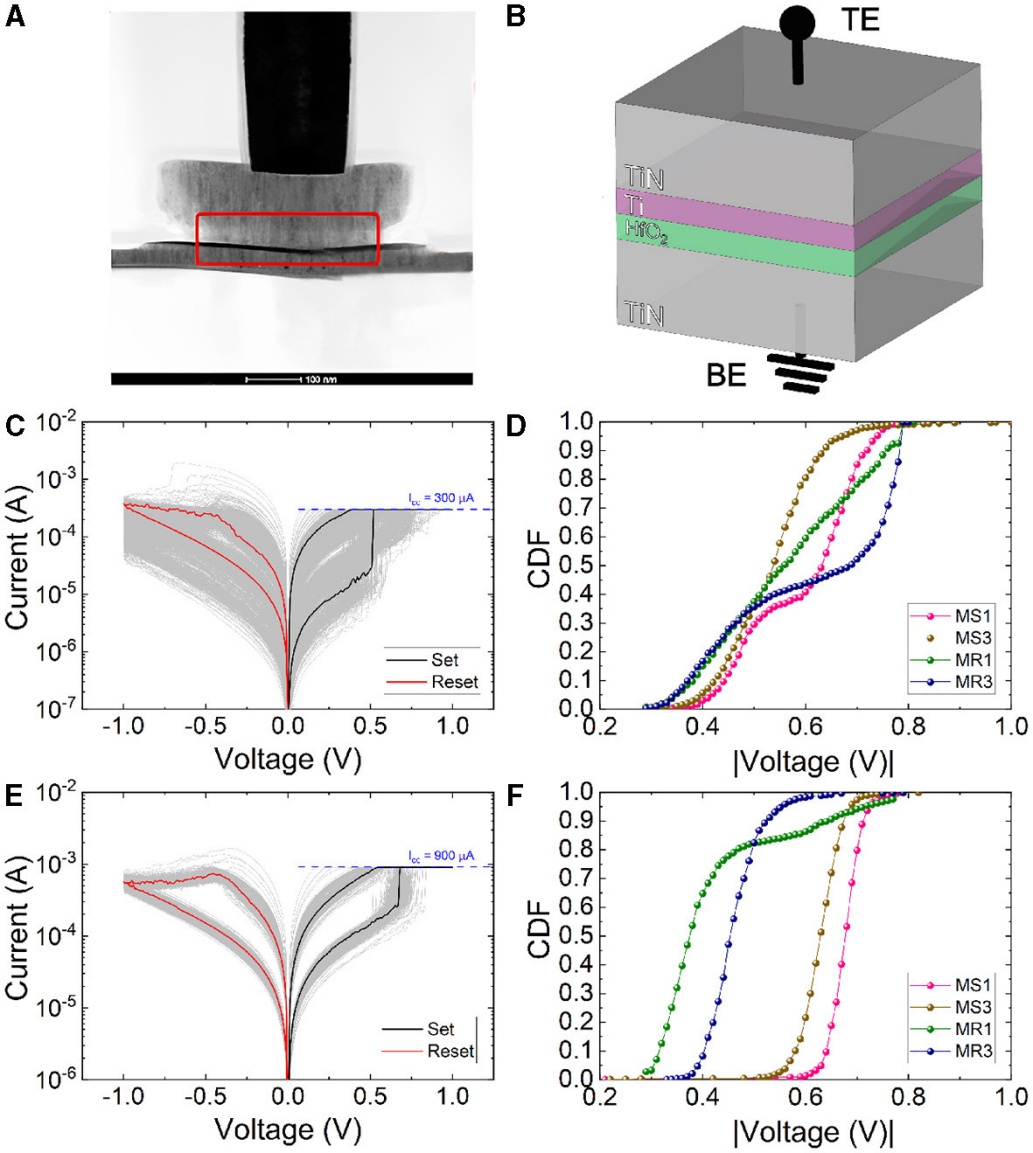
**(A)** Cross-sectional TEM image of a device fabricated on the metal line 2, **(B)** MIM stack schematics. **(C)** Experimental current vs. voltage curves for 1,000 consecutive RS cycles measured for a compliance current (I_CC_) = 300 μA. **(D)** V_set_ and V_reset_ cumulative distribution functions (CDFs) calculated for the extraction methods [MS1 and MS3 for the set voltage extraction; and MR1 and MR3 for the reset voltage extraction as explained in (Maldonado et al., 2022)] for the curves corresponding to **(C)**. **(E)** Experimental current vs. voltage curves for 1,000 consecutive RS cycles measured assuming a I_CC_ of 900 μA. **(F)** V_set_ and V_reset_ CDFs calculated as in (Maldonado et al., 2022) for the curves corresponding to **(E)**.

The devices employed here are single MIM structures placed on the metal line 2 of the CMOS process (130 nm technology) (Figure 1A). Each device is integrated within one of the 108 different dies included in the 200 mm wafer. Their size is 600x600 nm^2^. They are based on a TiN/Ti/HfO_2_/TiN stack (see Figure 1B), with a TiN bottom electrode (BE) which is 150 nm thick, an 8 nm HfO_2_ switching layer, a 7 nm oxygen scavenging layer made of titanium and a TiN (150 nm thick) top electrode (TE). The metal layers were deposited by magnetron sputtering, and the dielectric layer was grown by atomic layer deposition (ALD). The electrical measurements were performed by means of a Keysight B1500A semiconductor parameter analyzer connected to a probe station Karlsuss PSM6. The Keysight B1511B medium power source measurement unit (SMU) module was employed for quasi-static ramped voltage stress, and the Keysight B1530 module, a waveform generator and fast measurement unit provided the voltage pulse trains. The voltage signal was applied to the TE, while the BE was grounded. The semiconductor parameter analyzer was connected to a computer via GPIB and controlled using MATLAB.

We have plotted I–V curves measured as a long series (1,000 cycles) of successive set and reset processes. Different values of I_CC_ were employed in Figure 1C (I_CC_ = 300 μA) and Figure 1E (I_CC_ = 900 μA). In order to extract the most representative RS parameters such as the set and reset voltages and currents, different advanced numerical procedures are employed. The first method to determine the set voltage (MS1) consists in finding the maximum value of the numerical derivative (Maldonado et al., 2022). Another methodology [MS3 in Maldonado et al. (2022)] searches for the maximum separation of the experimental curve to an imaginary straight line that joins the first point in the measured curve and the first point where this current presents its maximum (it finds the set curve knee). Notice in Figures 1D, F that MS1 extracted values are higher than the MS3 ones, as found in Perez et al. (2023). For the reset voltage, we search for the current derivative minimum [MR1 in Maldonado et al. (2022)] and the current maximum [MR3 in Maldonado et al. (2022)]. The behavior of MR1 and MR3 extracted values is coherent to the one reported in Perez et al. (2023), as shown in Figures 1D, F.

In Supplementary Figures S1, S2 in the Supplementary material (SM), we show a thorough analysis of the set and reset processes in addition to a cycle-to-cycle variability study. The high resistance state (HRS) to low resistance state (LRS) resistance ratio is approximately 10 for the two I_CC_ under consideration, an appropriate value for memory applications. The variability for the set and reset voltages is low (Supplementary Figures S1C, S2C) although a better behavior is obtained in general for the high I_CC_ since a more stablished conductive filament is formed, and this allows a more uniform switching (Aldana et al., 2020a,b).

## 3. Results and discussion

We have analyzed different synaptic features in the devices under study to assess their appropriateness for neuromorphic engineering applications.

### 3.1. Potentiation and depression characteristics

**Figure 2:**
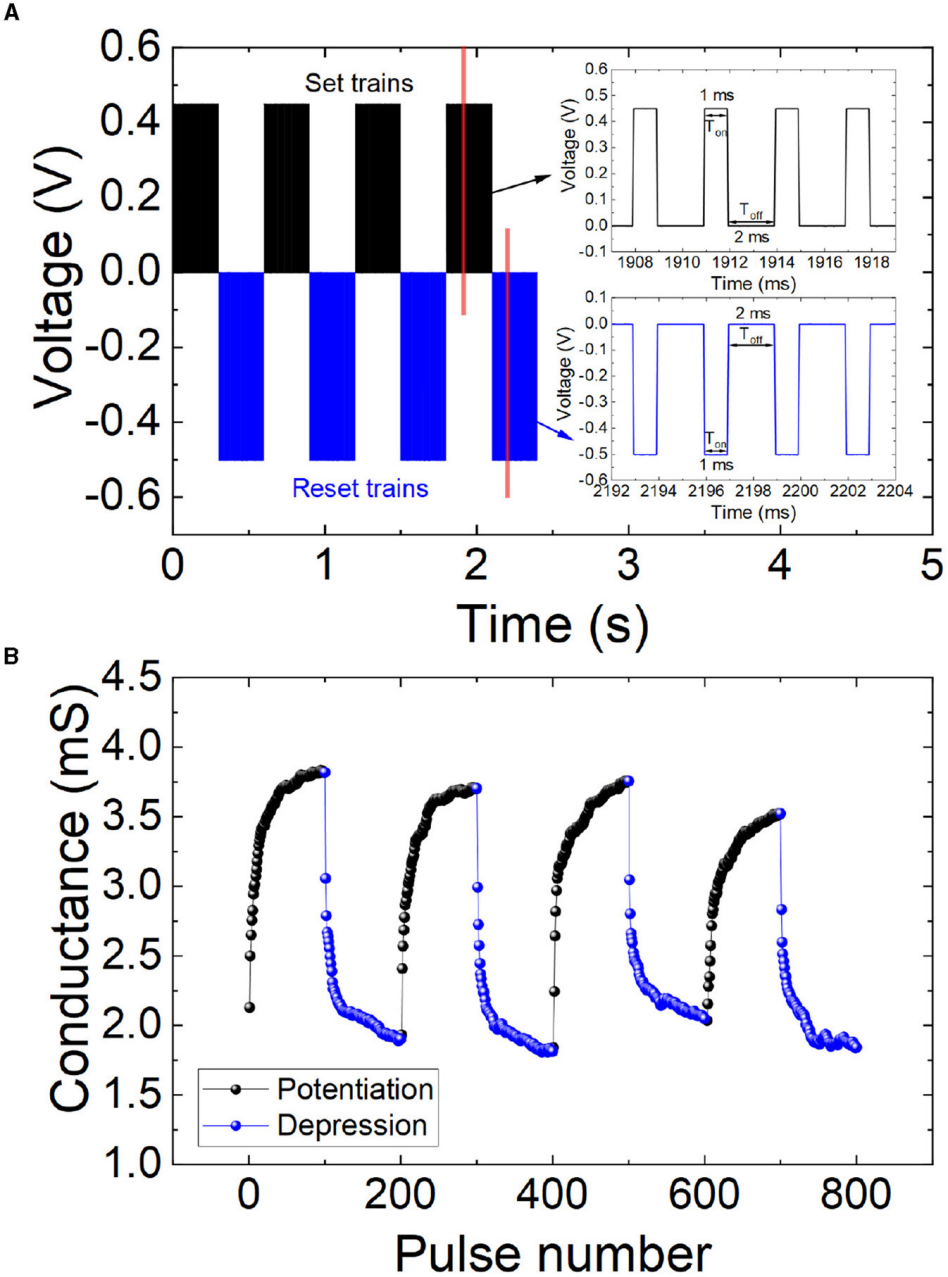
**(A)** Voltage vs. time for a series of applied signals consisting in positive and negative pulse trains. Set pulses are shown in black lines (0.45 V and a time length of 1 and 2 ms for T_on_ and T_off_ respectively), while reset pulses are plotted in blue lines (-0.5 V and a time length of 1 and 2 ms for T_on_ and T_off_ respectively) as depicted in the inset. **(B)** Conductance vs. pulse number (non-volatile states). The potentiation and depression effects can be easily observed. The input signals employed in these measurements are those described in **(A)**.

In order to correctly mimic biological synapses, the devices should show a controlled conductance variation. This means a modulation of the switching behavior (by means of gradual set and reset processes) to allow, in terms of ANN implementations, a regulated synaptic weight change. To do so, different voltage pulse trains can be employed. In particular, we used successive set (V_set_ = 0.45 V and fixed pulse widths, T_on_ = 1 ms, T_off_ = 2 ms, for a progressive set process that produces potentiation) and reset (V_reset_ = -0.5 V and fixed pulse widths, T_on_ = 1 ms, T_off_ = 2 ms, for a progressive reset process that leads to depression) pulse trains, as shown in Figure 2A. Multiple pulse widths and frequencies were employed in the measurements; in Figure 2, we just show the best results obtained. The voltage values employed are coherent with those found for the quasistatic I-V curves under ramped voltage stress (Figures 1C, E); in addition, they are in line with other previous works, see for instance Ismail et al. (2022). The memristive device response to successive pulse trains in terms of conductance is shown in Figure 2B for synaptic potentiation and depression.

**Figure 3:**
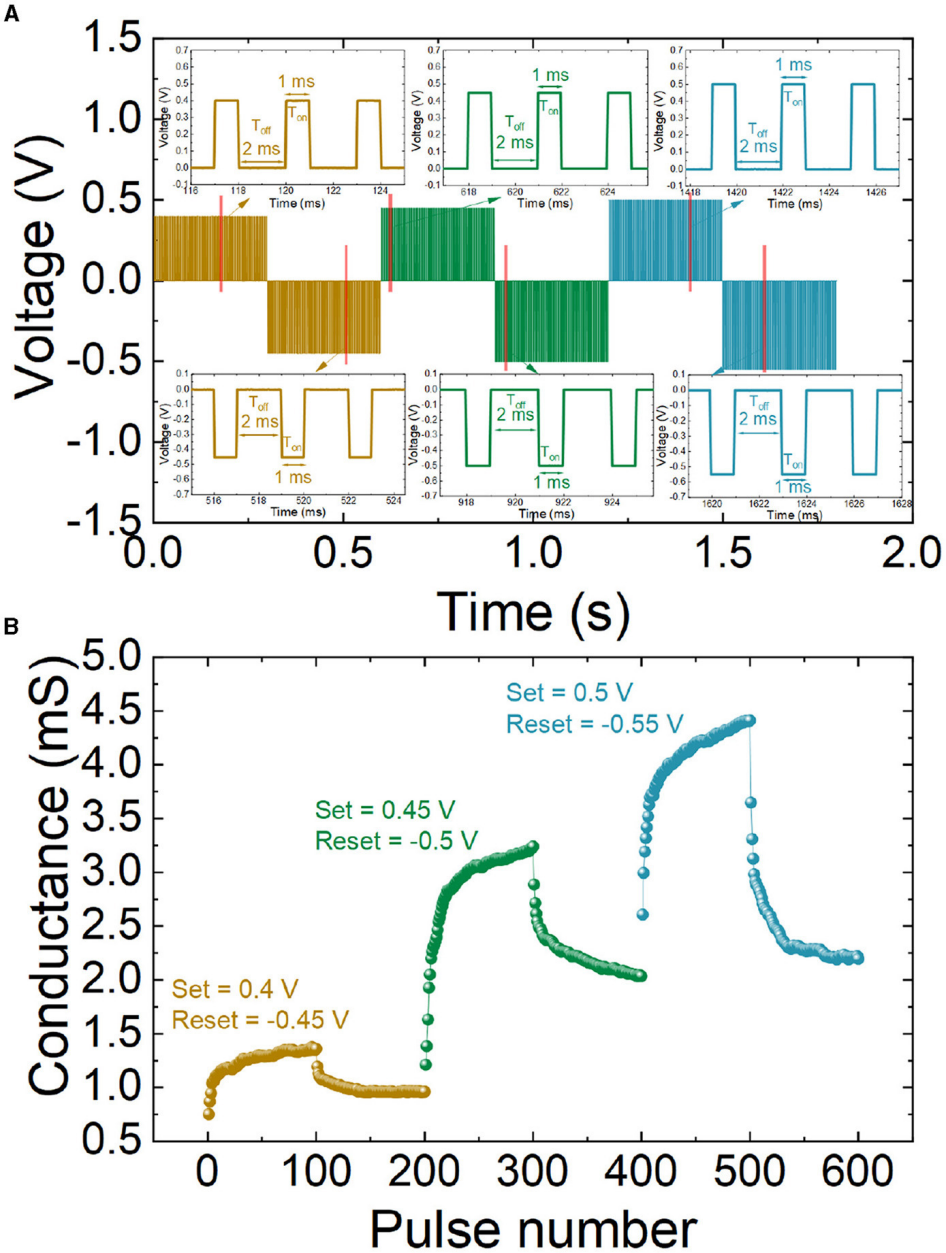
**(A)** Voltage vs. time series of positive and negative pulses applied to the device. Different amplitudes are considered, potentiation spikes range from 0.4 V, 0.45 V, and 0.5 V with a duration of 1 and 2 ms for T_on_ and T_off_, respectively, while depression spikes range from -0.45 V, -0.5 V, and -0.55 V with a duration of 1 and 2 ms for T_on_ and T_off_, respectively. See in the insets a zoomed-in part of the pulse series. **(B)** Synaptic plasticity, potentiation, and depression events (non-volatile states). Device conductance vs. pulse number making use of the pulse series described in **(A)**.

To further demonstrate the characteristics and the reproducibility obtained with potentiation and depression stimuli, three pulse series (to allow potentiation and depression cycles) were repeated for different amplitudes (0.4 V and -0.45 V for cycle 1; 0.45 V and -0.5 V for cycle 2; 0.5 V and -0.55 V for cycle 3), while the pulse widths are fixed to 1 ms (T_on_, when the pulse is active) and 2 ms (T_off_, when the pulse is zero), as displayed in Figure 3A. As highlighted above, in the context of neuromorphic engineering, the pulses resemble spikes, the communication signals at the neural level. During a sequence of potentiation spikes, the memristive conductance rises. Afterward, a sequence of depression spikes leads to a conductance reduction cycle, see Figure 3B.

### 3.2. Excitatory postsynaptic current

**Figure 4:**
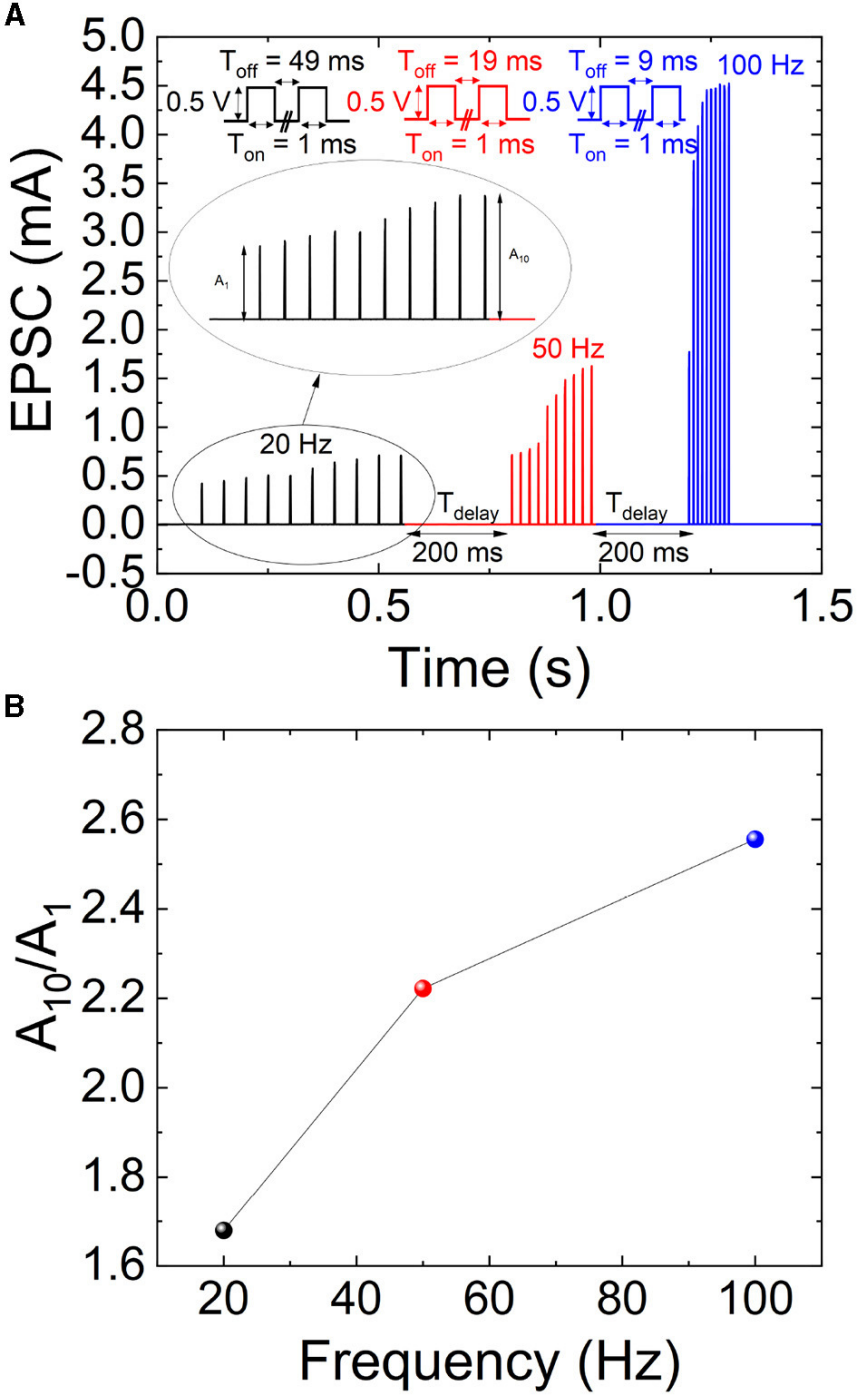
**(A)** EPSC response after a train of 10 spikes applied with 0.5 V amplitude for 1 ms. Different pulse train frequencies were employed (20, 50 and 100 Hz). **(B)** EPSC amplitude gain for A_10_/A_1_ vs. pulse train frequency.

The device excitatory postsynaptic current (EPSC) characterizes the synaptic response to applied stimuli with different frequencies. In particular, in our EPSC study, we employed 20 Hz, 50 Hz, and 100 Hz, see Figure 4A. The stimuli consist in a train of spikes with an amplitude of 0.5 V and a time length (T_on_) of 1 ms, while the time values between spikes (T_off_) are 49 ms for 20 Hz, 19 ms for 50 Hz, and 9 ms for 100 Hz, see the schemes in the insets of Figure 4A. Notice that between the different spike trains, corresponding to each frequency, a 200 ms delay has been included to minimize inertial effects; after this delay time, the device operational region is assumed to cool down in what is related to thermal effects (Roldán et al., 2021). Consequently, previous signals do not affect. The EPSC increases with the pulse train frequency. This effect is depicted in Figure 4A, and it is visualized as the gain ratio of the amplitudes corresponding to the last and the first spikes in the series. The higher the stimulus frequency, the higher the EPCS gain ratio (Figure 4B). Consequently, high-frequency inputs make the synapse more active, which is beneficial for high-pass filtering in the context of spiking neural networks (Ismail et al., 2022; Li et al., 2023). At this point, it is important to highlight that in spike processing, the dynamic adaptation of the synaptic weight gives rise to many significant pattern representation and processing capabilities (He et al., 2021). In this respect, features such as EPCS are key for correctly mimicking biological synapses by means of memristors.

### 3.3. Paired-pulse facilitation

**Figure 5:**
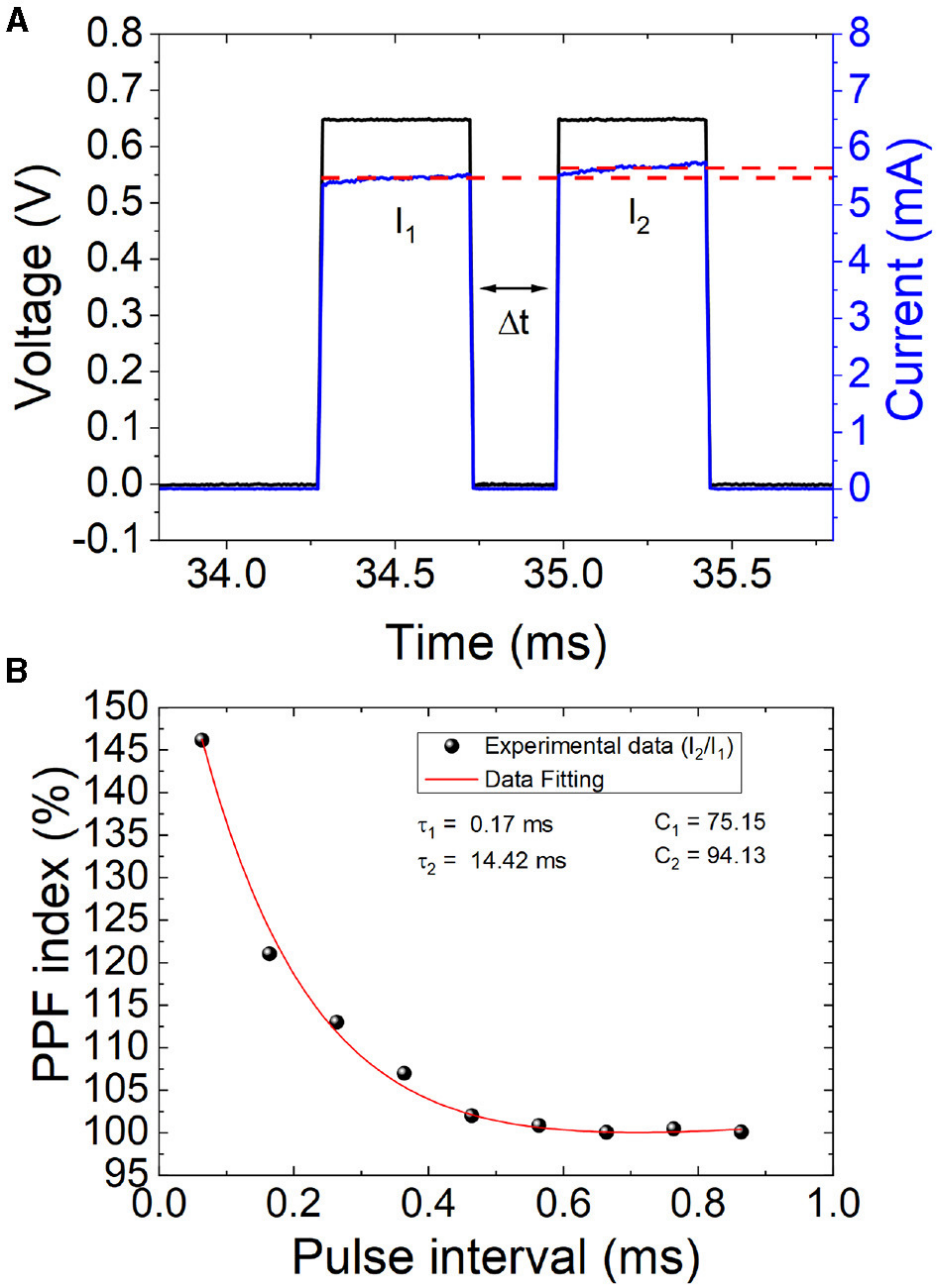
**(A)** Temporal PPF current response to two consecutive pulses with a set delay (Δ*t*) between spikes. **(B)** PPF index calculated as defined in Equation 1 vs. Δ*t*. The experimental data (black dots) have been fitted (red line) by means of Equation 2 with the following relaxation times and constants, τ_1_ = 0.17 ms, τ_2_ = 14.42 ms, *C*_1_ = 75.15 ms, *C*_2_ = 94.13 ms.

PPF occurs when two closely time-spaced spikes are applied to a neuron, causing the second pulse to produce a stronger response than the first. This effect is known as facilitation (Markram et al., 1997; Zucker and Regehr, 2002), and it is required for decoding temporal information in biological synapses and increasing the selectivity and information capacity of neural circuits (Zucker and Regehr, 2002). For its importance in neural processing, allowing neurons to encode data more efficiently by increasing the strength of synaptic connections between them, we have considered PPF in our analysis. We introduce two consecutive spikes (pulses generated with the semiconductor parameter analyzer, Figure 5A, in the set process operation regime of the cell), with a set delay in between, to study the corresponding synaptic response (a typical short-term synaptic plasticity effect). As explained, the first spike induces a postsynaptic response and the second induces a larger reaction. The interpulse time interval, Δ*t*, was employed as the key variable; the shorter this interval, the higher the ratio between the average current measured for the first (*I*_1_) and second (*I*_2_) spikes (see Figure 5B).

Equation 1 calculates a PPF index in the usual way (Ismail et al., 2022):


(1) $$\begin{array}{l} PPFindex=\frac{I_{2}}{I_{1}}\cdot 100 \end{array}$$

Moreover, a curve can be employed to fit PPF experimental data that show an exponential dependence with the interspike time (Zucker and Regehr, 2002) (Equation 2),


(2) $$\begin{array}{l} PPFindex=C_{1}\cdot exp\left(\frac{-\Delta t}{\tau_{1}}\right)+C_{2}\cdot exp\left(\frac{\Delta t}{\tau_{2}}\right) \end{array}$$

where τ_1_ and τ_2_ are both relaxation times, and *C*_1_ and *C*_2_ are fitting constants. In particular, for our data (Figure 5B), the following values work correctly for the fitting: τ_1_ = 0.17 ms; τ_2_ = 14.42 ms, *C*_1_ = 75.15 and *C*_2_ = 94.13.

For our data, a simplified version of Equation 2 could work with just three parameters (τ_1_, *C*_1_ and *C*_2_). However, the two times constant are needed if fast and slow decaying terms need to be modeled (Wang et al., 2015). See that a gradual decrease of the PPF index is obtained as the spike intervals increases. From the viewpoint of the physical mechanisms involved in the switching operation of the devices, a shorter interpulse time involves a higher temperature in the active region of the dielectric when the second spike comes in. Taking into account that the physical mechanisms behind switching are thermally activated (Dirkmann et al., 2018; Aldana et al., 2020a), the effects of the second spike in taking the set process further, and increase the device current, are more effective.

### 3.4. Spike timing dependent plasticity and SNN analysis

As highlighted previously, STDP is an important synaptic feature that allows the incorporation of a learning rule in spiking neural networks (Roldan et al., 2022). It can be used to implement associative learning in SNNs. Competition of spike-conducting pathways plays an essential role in establishing associations of neural connections; on the network scale, STDP potentiates the shortest neural pathways and depresses alternative longer pathways (Lobov et al., 2020). It describes the adjustment of the connection strength between neurons based on the time relation between the postsynaptic neuron and presynaptic neuron spikes in a particular synapsis (Roldan et al., 2022; Zhu et al., 2023), this mechanism is key for synaptic plasticity in biological neural circuits.

**Figure 6:**
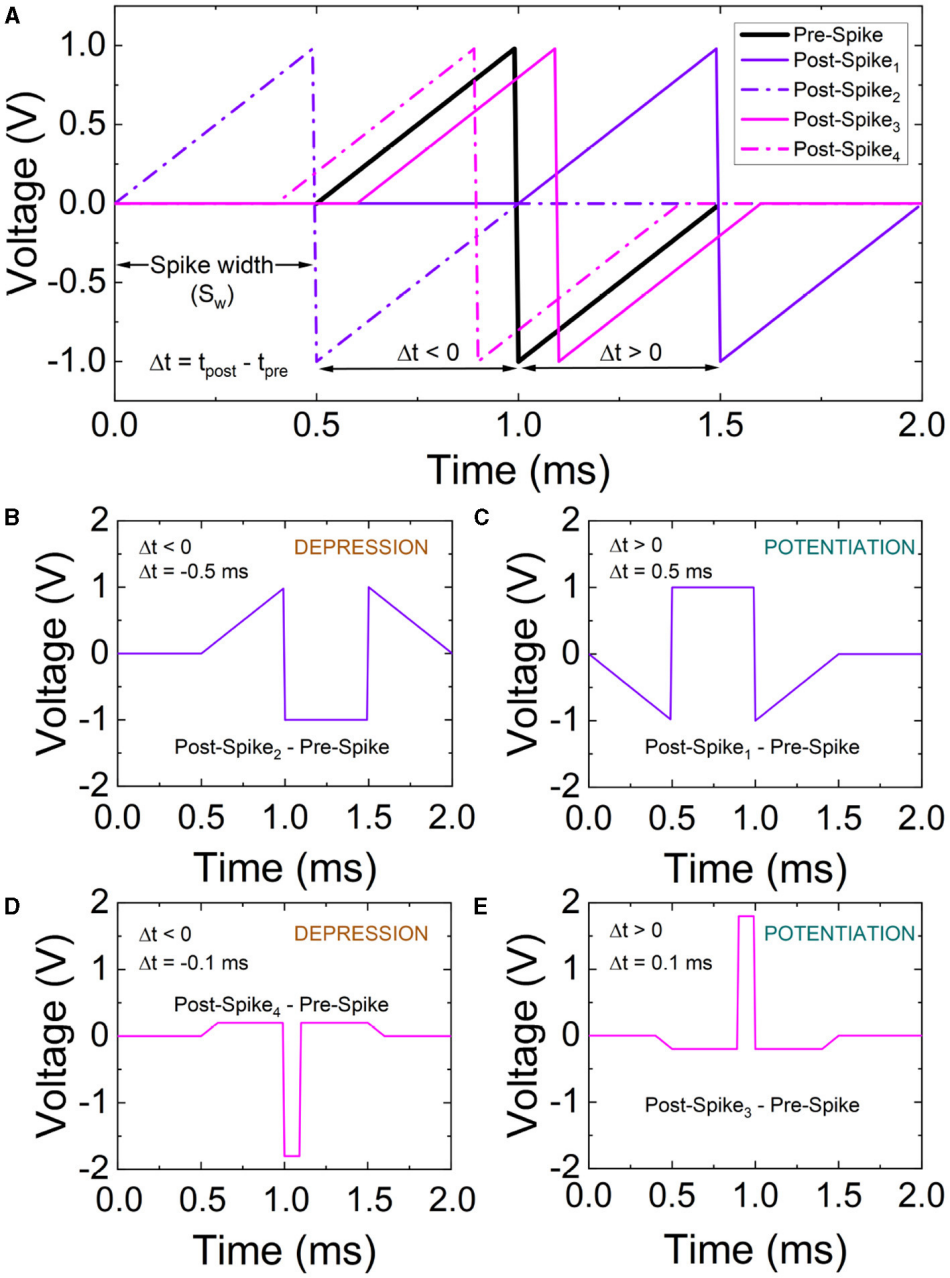
**(A)** Time scheme of the spikes employed for the STDP characterization. To simplify the measurement process, the post-spike and pre-spike are subtracted resulting in the signals in **(B–E)**. For the measurements we assume distinct spike widths (S_w_) with varying delays (Δ*t* = *t*_*post*_ − *t*_*pre*_).

STDP characterization in memristive devices consists in the application of a delayed pair of voltage spikes to the electrodes (Roldan et al., 2022). In our experiments, the shape of the applied pulses is displayed in Figure 6A. The timing of the spikes at the top and bottom electrodes is referred to as *t*_*pre*_ and *t*_*post*_, with the delay between them as Δ*t* = *t*_*post*_−*t*_*pre*_. The bottom electrode can be left grounded to ease the measurement process and an input signal obtained subtracting the post and pre-spikes is used at the top electrode (Figures 6B–E).

**Figure 7:**
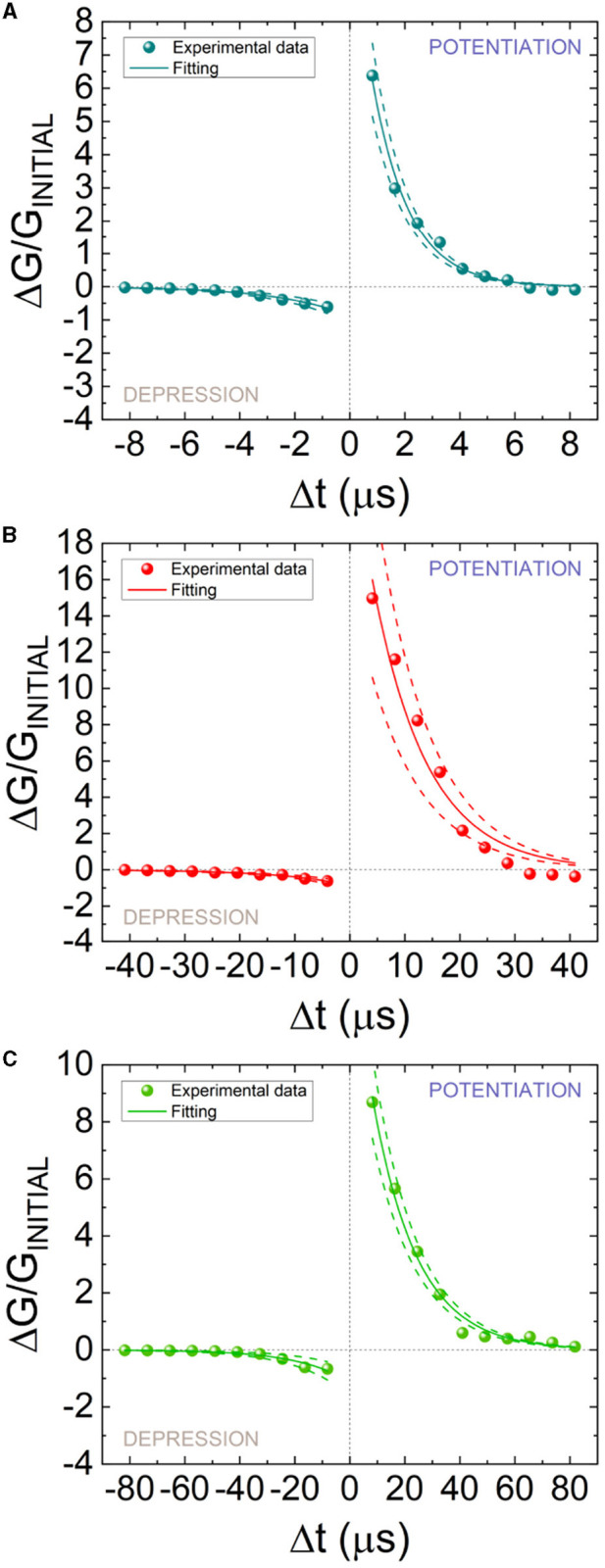
STDP measurements (symbols) vs. pre- and post-spike delay for different spike time widths (S_w_) **(A)** 10, **(B)** 50, and **(C)** 100 μs. A fitting procedure has been performed using Equation 3 to reproduce the experimental data (solid lines). The dashed lines are fitted to encompass the experimental data distributions while retaining the same time constant parameters (τ+, τ−) in Equation 3 for the depression or potentiation curves. The fitting constants of the STPD data are given in Table 1.

In Figure 7, STDP measurements are shown. The change in device conductance (Δ*G*) was determined based on the starting conductance (*G*_*INITIAL*_) which was obtained at the beginning of the measurement process. A good STDP behavior is obtained for different spike time widths (S_w_), namely, 10, 50, and 100 μs. In order to implement the STDP as a learning rule for SNNs, Equation 3 is employed to fit the experimental data (Ismail et al., 2022; Roldan et al., 2022). *A* and τ parameters for potentiation and depression are employed for the experimental data fitting.


(3) $$\left\{ \begin{array}{l} \frac{\Delta G}{G_{INITIAL}}=A_{+}exp\left(\frac{-\Delta t}{\tau_{+}}\right)\text{ for }\Delta t>0\\ \frac{\Delta G}{G_{INITIAL}}=-A_{-}exp\left(\frac{\Delta t}{\tau_{-}}\right)\text{ for }\Delta t<0 \end{array}\right.$$

The fitting (the parameters are listed in Table 1) of the whole set of experimental data is shown in solid lines, while two other fittings to encompass the experimental dataset are depicted in dashed lines.

**Table 1:** Parameters to reproduce the STDP measurements in Figures 7A–C for different spike time widths (Equation 3).

**Line**	**Fitting**	**Potentiation**			**Depression**		
**fitted**	**variable**	**10** μ**s**	**50** μ**s**	**100** μ**s**	**10** μ**s**	**50** μ**s**	**100** μ**s**
Top	*A*_+_/*A*_−_	13.56	32.36	17.10	0.67	0.68	0.64
(dashed line)	τ_+_/τ_−_ (μs)	1.33	9.86	16.18	2.57	12.41	18.3
Average	*A*_+_/*A*_−_	11.53	24.20	14.70	0.88	0.87	1.14
(solid line)	τ_+_/τ_−_ (μs)	1.33	9.86	16.18	2.57	12.41	18.3
Bottom	*A*_+_/*A*_−_	9.50	16.04	12.31	1.11	1.06	1.65
(dashed line)	τ_+_/τ_−_ (μs)	1.33	9.86	16.18	2.57	12.41	18.3

**Figure 8:**
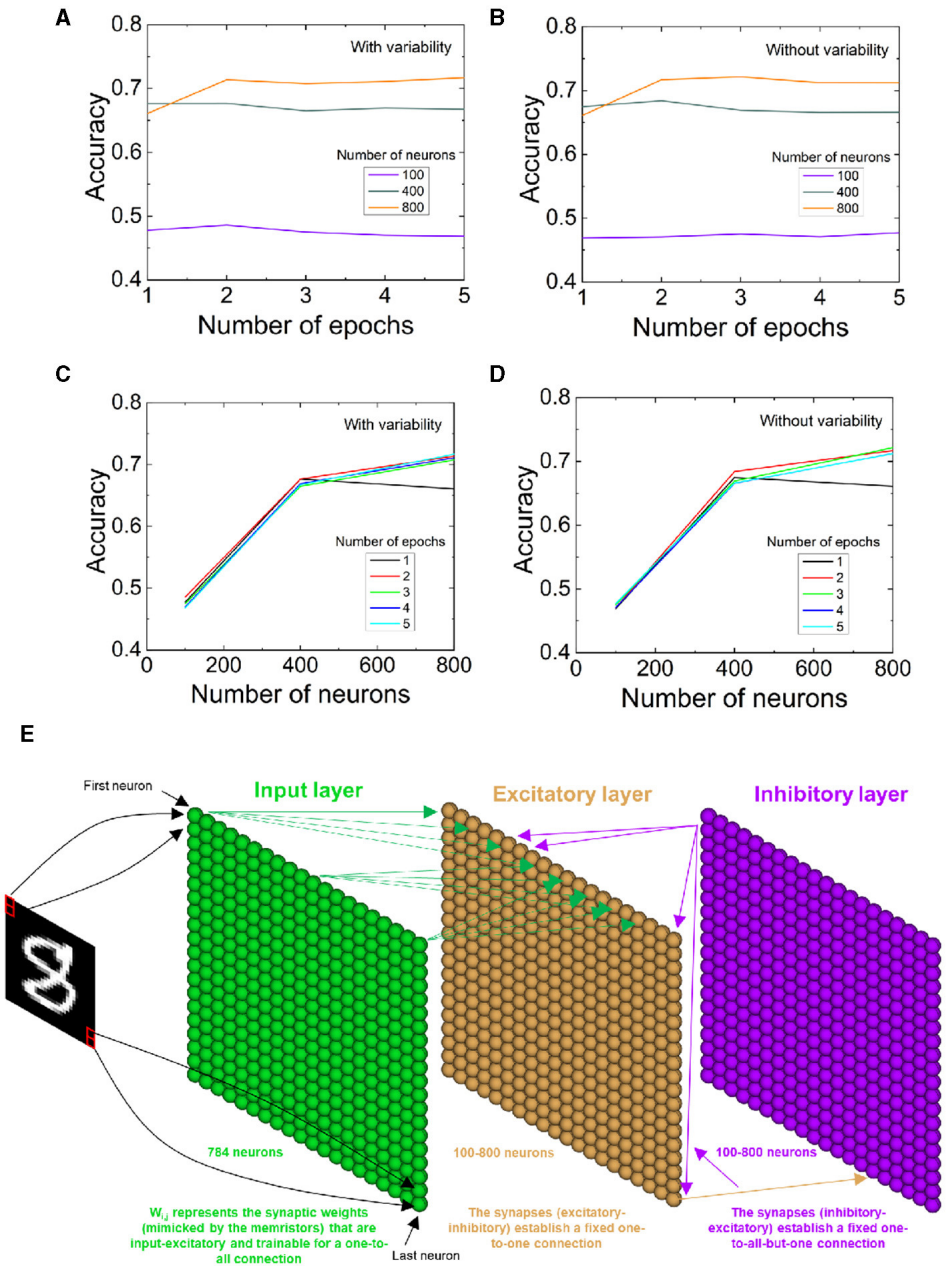
**(A, B)** SNN recognition accuracy vs. number of epochs for different number of neurons including (without) variability in the parameters of the STPD data fitting. **(C, D)** SNN recognition accuracy vs. number of neurons for different number of epochs including (without) variability. **(E)** SNN architecture schematics.

We have made use of the device characteristics analyzed here to build a SNN. The network architecture is shown in Figure 8E, and the operational features are given in the supplementary note 1 in the SM. The input image dataset was considered from the Modified National Institute of Standards and Technology (MNIST). The MNIST dataset is formed by 28 × 28 grayscale pixel images that consist of 70,000 handwritten digits labeled in the interval [0, 9], divided into a training set (60,000 images) and a test set (10,000 images).

We utilize the parameters shown in Table 1 coming from the fitting of Figure 7 STDP data to determine the SNN learning rule with an unsupervised learning scheme. The network input layer consists of 784 neurons, and it has been adapted to the dataset chosen in this case. Variability (as can be calculated with the constants of Table 1) was incorporated in the equation that determines the synaptic weight (Roldan et al., 2022) [traces are employed, whose value is linked to spike magnitude, the time constants in Table 1 are introduced in the differential equations corresponding to the neuron model, in our case the leaky-integrate and fire, and the *A*_+_ and *A*_−_ constants are employed in the equations that lead to the synaptic weight calculation (Roldan et al., 2022)]. With the new differential equation for the synaptic weight determination, including variability, we repeated the training process. Once the SNN pieces were put together, we analyzed the recognition accuracy considering a different number of epochs (Figures 8A, B) as well as a different number of neurons (Figures 8C, D). Notice that the higher the number of neurons, the better recognition accuracy for the MNIST dataset; nevertheless, the recognition accuracy improvement with the number of neurons diminish for values above 400. The inclusion of variability mostly affects the SNN accuracy with a low number of neurons; nonetheless, for 400, and mostly for 800 neurons, variability influence is low due to the SNN stochastic nature. In fact, for the higher number of neurons employed (800) and the higher number of epochs (5), there is no difference when variability is included in the calculation of the synaptic weights (see Figures 8A–D). In some experiments, higher accuracy values are obtained including variability.

### 3.5. Stochastic resonance

The SR measurements were performed using a ramped input signal (0.28 V/s) and adding input noise with a null mean and different standard deviations (σ) (Supplementary Figure S3 in the SM). Furthermore, for the experimental SR analysis, three different statistical distributions where employed: normal or Gaussian, uniform and exponential (Heumann et al., 2016). A total of 100 I-V complete RS curves were obtained, as in Figure 1, for each standard deviation and statistical distribution. See the whole evolution of R_ON_ and R_OFF_ in the measurements in Supplementary Figure S4 (SM). There is a clear variation in the resistance evolution with rising, as expected. In particular, for the normal distribution, the variation is higher, for the exponential distribution the change in R_ON_ and R_OFF_ is found in between the results for the normal and uniform distributions. In what is connected to the set and reset voltages, notice in Supplementary Figure S5 that the added noise does not disturb much the RS operation. This result is due to the inherent stochasticity of RS operation that is resilient to added random noise. As the noise intensity rises, the difference between the set and reset voltages slightly shrinks for the three statistical distributions under study, this difference is higher for the normal distribution. As expected, the variation of the set and reset voltages increases as the noise standard deviation rises.

**Figure 9:**
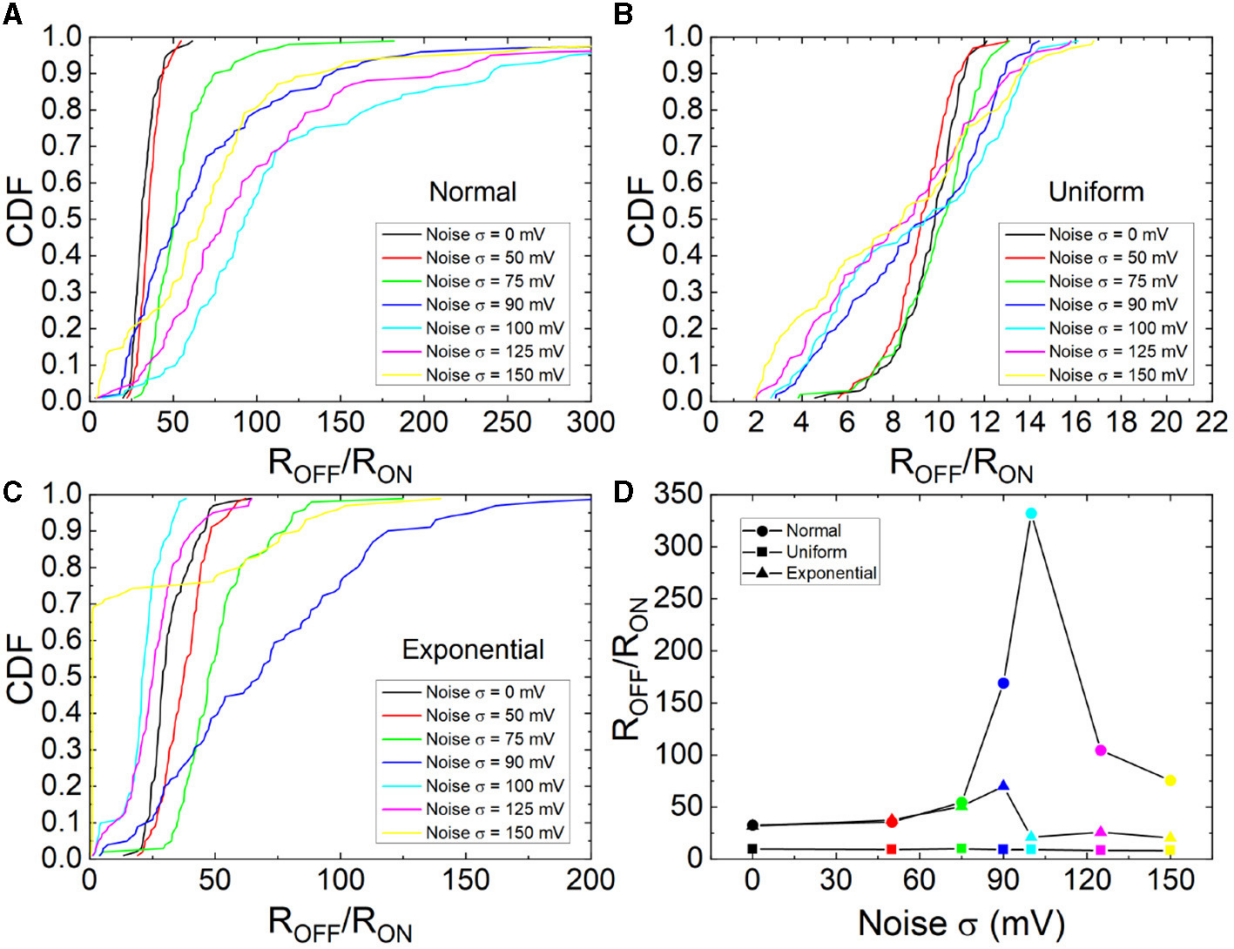
CDFs for the R_OFF_/R_ON_ ratio of the measured RS cycles assuming several values and types of noise, **(A)** normal, **(B)** uniform, **(C)** exponential. **(D)** Mean R_OFF_/R_ON_ ratio vs. noise standard deviation (100 values were considered for each symbol shown).

The cumulative distribution functions of the R_ON_ and R_OFF_ ratio are shown for different σ values and different statistical distributions in Figure 9. The CDFs shift to higher values as the noise intensity rises till approximately σ = 100 mV; at this point, the CDFs shift back. In this respect, an improvement of the device response is obtained by means of the addition of noise; in particular, at the σ value, where the resonance takes place. This behavior is clear for the normal distribution although it is not straight forward for the exponential and uniform distributions case.

The mean R_OFF_/R_ON_ ratios vs. noise intensity was plotted in Figure 9D. A clear SR behavior is seen as it was highlighted in Mikhaylov et al. (2021) and Cirera et al. (2022). This result is in line with those shown in Rodriguez et al. (2022) although the technology employed in the study is different. In our case, SR depends on the statistical distribution function employed to generate the input noise.

## 4. Conclusion

TiN/Ti/HfO_2_/TiN memristive devices have been fabricated and experimentally characterized. The main features to make them work by mimicking biological synapses are studied in the context of neuromorphic computing. Different models are included to reproduce experimental data. Among other effects, spike timing dependent plasticity data are obtained in the laboratory and modeled to be employed as the learning rule to implement a spiking neural network to recognize the numerical MNIST dataset. The SNN was trained with and without variability in the STDP data. It has been shown that variability influences on the network recognition accuracy although the increase of the number of neurons and training epochs can help to compensate. Finally, stochastic resonance is studied as another synaptic feature. It is shown that this effect is important and greatly depends on the noise statistical characteristics.

## Data availability statement

The raw data supporting the conclusions of this article will be made available by the authors, without undue reservation.

## Author contributions

DM: Investigation, Writing—review and editing. AC: Investigation, Writing—review and editing. EP: Writing—review and editing. RR-Z: Investigation, Writing—review and editing. EP-B: Investigation, Writing—review and editing. MK: Investigation, Writing—review and editing. FJ-M: Writing—review and editing. CW: Writing—review and editing. JR: Conceptualization, Writing—original draft.
