# Supplementary material for: TiN/Ti/HfO2/TiN memristive devices for neuromorphic computing: from synaptic plasticity to stochastic resonance
Source: Front Neurosci. 2023 Sep 19;17:1271956. doi: 10.3389/fnins.2023.1271956 (PMC10546015; doi:10.3389/fnins.2023.1271956)
Supplement: Supplementary file 1 [file Data_Sheet_1.PDF]

Supplementary Material

1 SUPPLEMENTARY NOTE 1

The SNN is implemented using BindsNet (Hazan et al., 2018) (a Python library built on top of PyTorch (Paszke et al., 2017)). The network model can be executed on CPU or GPU. Several types of neurons, input encoding and learning methods (e.g. STDP) are implemented to be chosen to configure different SNN options. See the scheme of the SNN architecture in the main manuscript (Figure 8e). The description of the neuron models employed in our network, and the implementation of the learning rule is given in Ref. (Roldan et al., 2022).

Each input image is presented (in the training process) for 350 ms to the SNN in the form of Poisson-distributed spike trains (firing rates are proportional to the intensity of the pixels of the MNIST images, with firing rates between 0 (black pixel) and 128 Hz (white pixel)). We disable the post-synaptic spike when training is over, establish each neuron spiking threshold, and make a class correspond to each neuron. This latter task is based on its highest response to the ten classes of digits over one presentation of the training set. For the synaptic weights training process, we do not employ labels. Finally, the class-assigned neurons response is employed to evaluate the SNN classification accuracy on the MNIST test set. The determination of the predicted digit is performed by averaging each neuron responses per class, and then choosing the class with the highest average firing rate.

The excitatory layer (for data processing) contains the same number of neurons than the layer with the inhibitory neurons. The neurons of the input layer are connected in an all-to-all fashion to the excitatory neurons (see arrows in Figure 8e). The excitatory neurons of the processing layer connect one-to-one to inhibitory neurons. However, each inhibitory neuron connects to all excitatory neurons excepts to the one from which it receives connection. This architecture allows lateral inhibition that leads the excitatory neurons to compete (see Figure 8e) (Diehl and Cook, 2015). The STDP data in Figure 7 were normalized to let potentiation and depression values be found in the similar interval.

2 SUPPLEMENTARY FIGURES

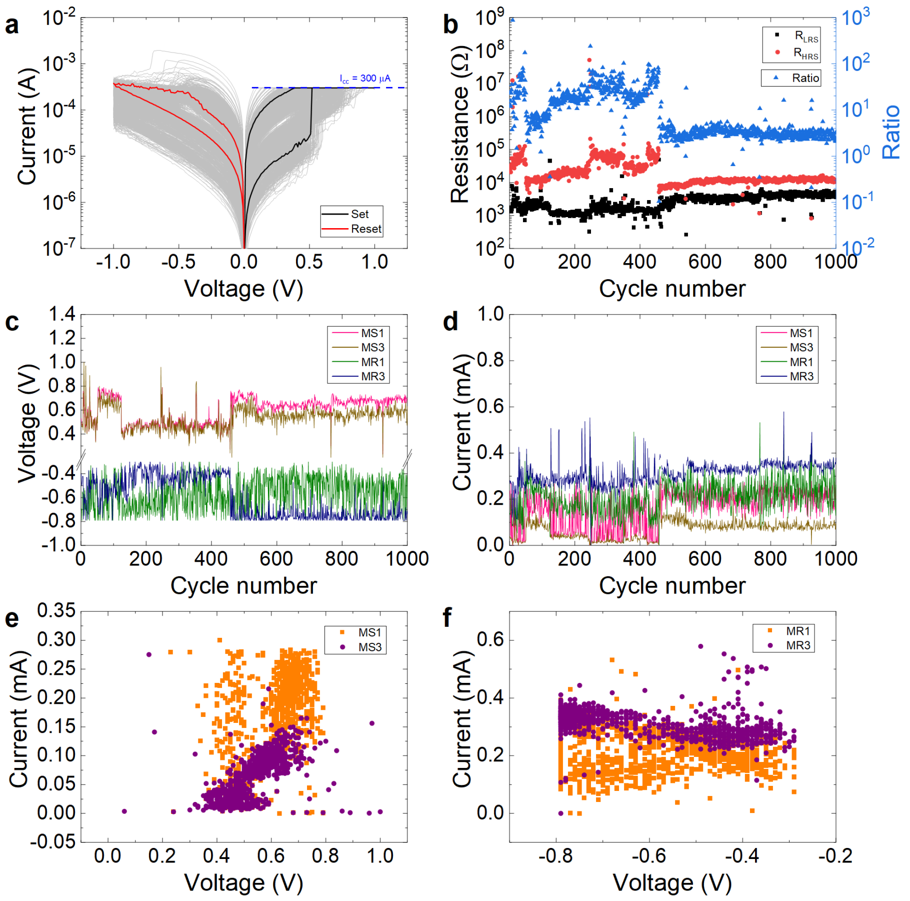

Figure S1. a Experimental current versus voltage curves for 1000 consecutive RS cycles measured for $I_{CC} = 300\ \mu A$. b LRS and HRS resistance (measured at 0.2 V) and corresponding ratio versus cycle number for the measured RS series obtained. c Set and reset voltages versus cycle number including the four numerical techniques employed, d current versus cycle number corresponding to the voltages extracted in c. e I_{set} versus V_{set} extracted employing methods MS1 and MS3 (Maldonado et al., 2022a,b), f I_{reset} versus V_{reset} extracted switching voltage parameters employing methods MR1 and MR3 (Maldonado et al., 2022a,b). The MS1 technique consists in finding the I-V curve point where the current derivative is maximum (Maldonado et al., 2022b); the MS3 procedures consists in finding the maximum separation from a straight line that joins the first and end points in a set curve (Maldonado et al., 2022b). The MR1 technique consists in finding the I-V curve point where the current derivative is minimum (Maldonado et al., 2022b); the MR2 technique consists in finding the I-V curve point where the current decrease in two consecutive points is maximum (Maldonado et al., 2022b).

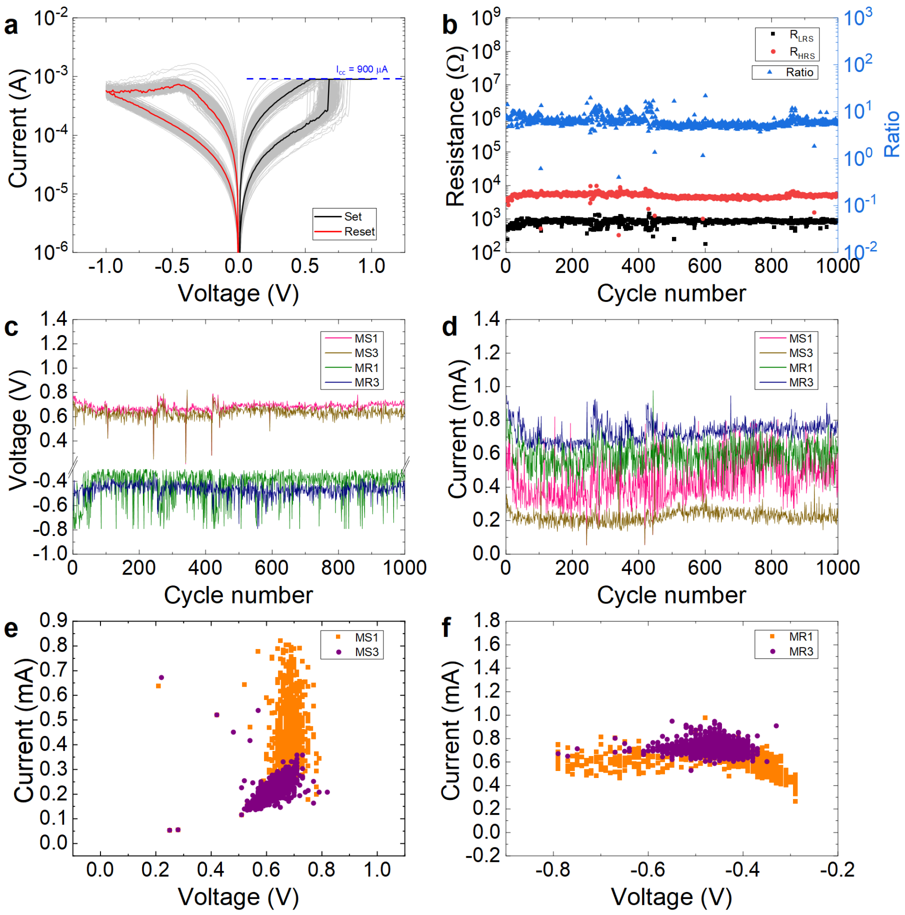

Figure S2. a Experimental current versus voltage curves for 1000 consecutive RS cycles measured for $I_{CC} = 900\ \mu A$. b LRS and HRS resistance (measured at 0.2 V) and corresponding ratio versus cycle number for the measured RS series obtained. c Set and reset voltages versus cycle number including the four numerical techniques employed, d current versus cycle number corresponding to the voltages extracted in c. e I_{set} versus V_{set} extracted employing methods MS1 and MS3, f I_{reset} versus V_{reset} extracted switching voltage parameters employing methods MR1 and MR3.

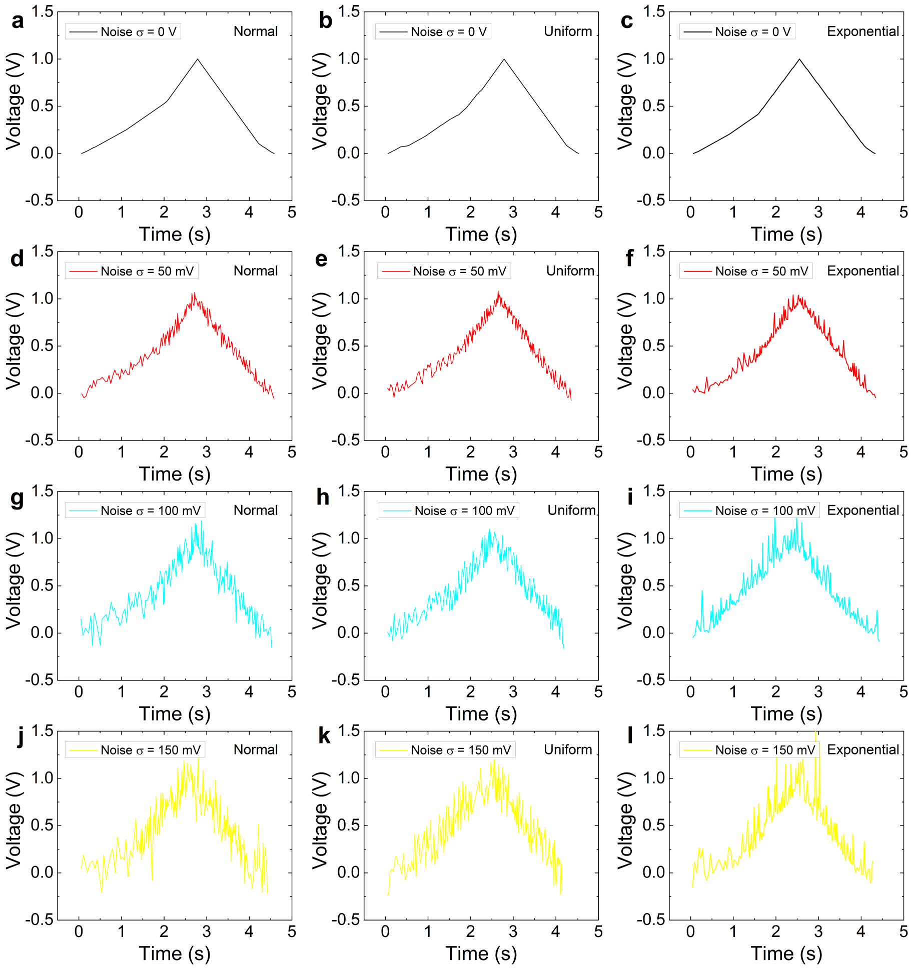

Figure S3. Voltage input signals employed in the SR measurements. Normal distribution: a no noise; d $\sigma = 50$ mV; g $\sigma = 100$ mV; j $\sigma = 150$ mV. Exponential distribution: b no noise; e $\sigma = 50$ mV; h $\sigma = 100$ mV; k $\sigma = 150$ mV. Uniform distribution: c no noise; f $\sigma = 50$ mV; i $\sigma = 100$ mV; l $\sigma = 150$ mV.

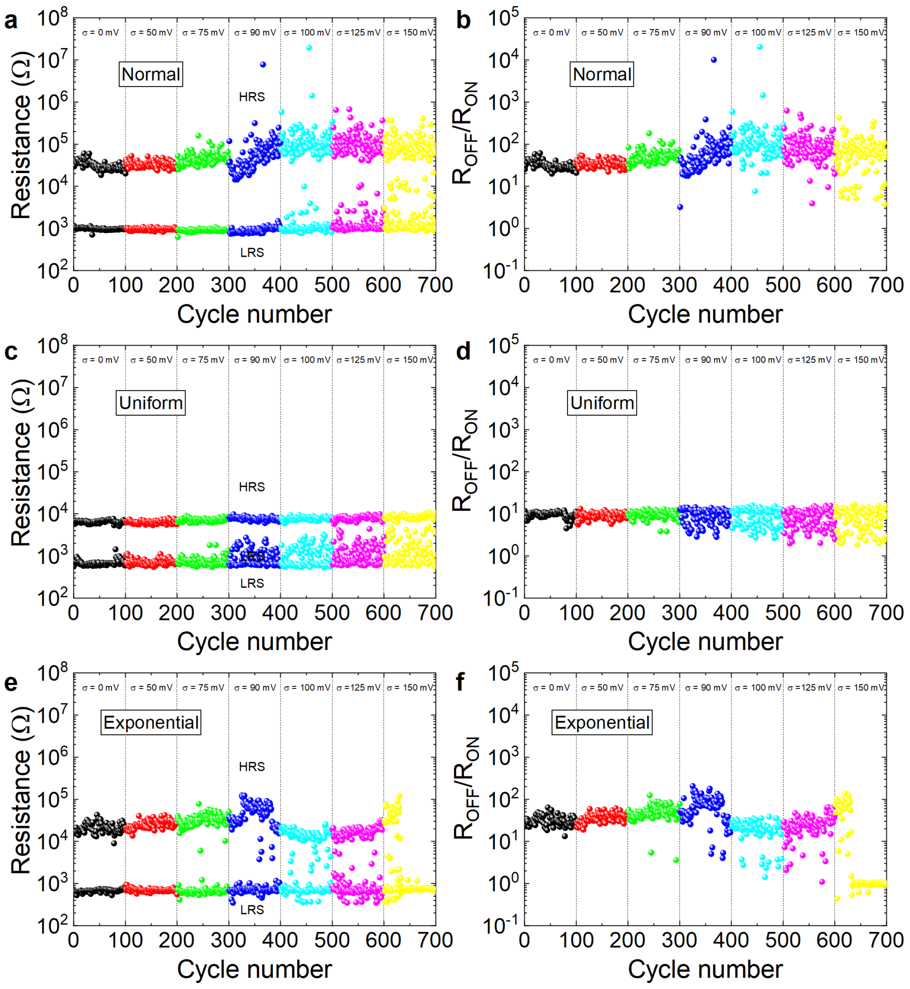

Figure S4. LRS and HRS resistance versus cycle number for RS series measured, the values were obtained at 0.2 V and correspond to different noise types: a normal or Gaussian, c uniform, e exponential. HRS/LRS resistance ratio versus cycle number calculated for the data corresponding to figures a, c and e for the noise generated with b normal or Gaussian, d uniform, and f exponential statistical distributions.

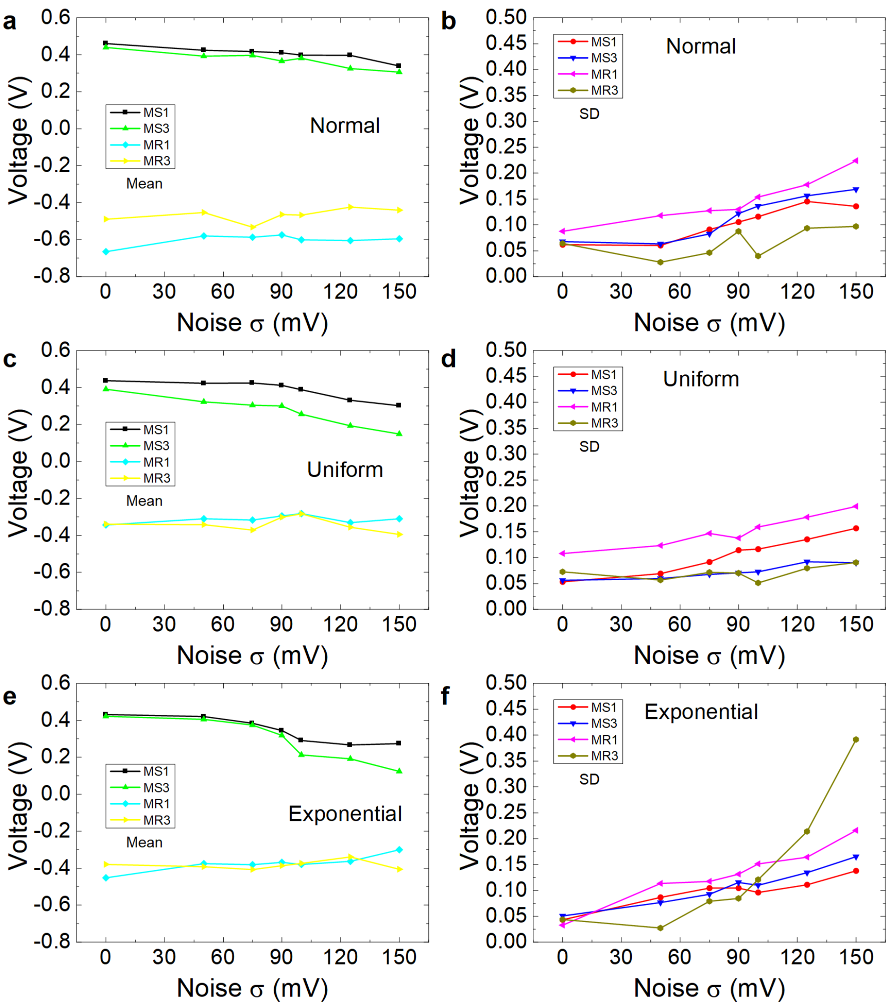

Figure S5. Mean values of the experimental set and reset voltages for the numerical techniques explained in Section 2 (Maldonado et al., 2022a,b) in the main manuscript versus noise level for the different types of statistical distributions: a normal or Gaussian, c uniform, e exponential. Standard deviation of the experimental set and reset voltages for the extraction techniques employed versus noise level for the different types of statistical distributions: b normal or Gaussian, d uniform, f exponential.

REFERENCES

Diehl, P. U. and Cook, M. (2015). Unsupervised learning of digit recognition using spike-timing-dependent plasticity. Frontiers in computational neuroscience 9, 99. doi:10.3389/fncom.2015.00099

Hazan, H., Saunders, D. J., Khan, H., Patel, D., Sanghavi, D. T., Siegelmann, H. T., et al. (2018). Bindsnet: A machine learning-oriented spiking neural networks library in python. Frontiers in neuroinformatics 12, 89. doi:10.3389/fninf.2018.00089

Maldonado, D., Aldana, S., González, M. B., Jiménez-Molinos, F., Campabadal, F., and Roldán, J. B. (2022a). Parameter extraction techniques for the analysis and modeling of resistive memories. Microelectronic Engineering 265, 111876. doi:10.1016/j.mee.2022.111876

Maldonado, D., Aldana, S., González, M. B., Jiménez-Molinos, F., Ibáñez, M. J., Barrera, D., et al. (2022b). Variability estimation in resistive switching devices, a numerical and kinetic monte carlo perspective. Microelectronic Engineering 257, 111736. doi:10.1016/j.mee.2022.111736

Paszke, A., Gross, S., Chintala, S., Chanan, G., Yang, E., DeVito, Z., et al. (2017). Automatic differentiation in pytorch. In NIPS 2017 Workshop on Autodiff

Roldan, J. B., Maldonado, D., Aguilera-Pedregosa, C., Moreno, E., Aguirre, F., Romero-Zaliz, R., et al. (2022). Spiking neural networks based on two-dimensional materials. npj 2D Materials and Applications 6. doi:10.1038/s41699-022-00341-5
